# Trends of temperature and total precipitable water, as well as the trend of surface pressure induced by CO_2_

**DOI:** 10.1038/s41598-024-80685-8

**Published:** 2024-11-25

**Authors:** Quanhua Liu, Christopher Grassotti, Yan Zhou, Yong-Keun Lee, Shuyan Liu, John Xun Yang

**Affiliations:** 1 Center for Satellite Applications and Research, National Environmental Satellite Data, and Information Service (NESDIS), National Oceanic and Atmospheric Administration, College Park, MD 20740 USA; 2 Earth System Science Interdisciplinary Center, University of Maryland, Maryland, 20740 USA; 3 Cooperative Institute for Research in the Atmosphere, Colorado State University, Fort Collins, CO 80523 USA

**Keywords:** Trends, Surface pressure, Surface temperature, TPW, ERA5, Climate sciences, Environmental sciences

## Abstract

Two total precipitable water (TPW) monthly time series derived from hourly analyses and from 1:30 am and 1:30 pm (local time) analyses, respectively, from European Centre for Medium-Range Weather Forecasts Reanalysis model version 5 (ERA5) data are practically identical. The TPW is increasing as surface and lower tropospheric temperatures are warming. Surface pressure is rising as well because of increased mass from atmospheric carbon dioxide and TPW above the surface. The close similarity of trends using hourly and twice-daily analyses suggest that using well-calibrated observations from a single sun-synchronous polar orbiting satellite, such as Suomi-NPP Advanced Technology Microwave Sounder measurements, can be sufficient to characterize global trends in climate-sensitive surface temperatures and TPW.

**Supplementary Information:**

The online version contains supplementary material available at 10.1038/s41598-024-80685-8.

## Introduction

Rising concentrations of atmospheric carbon dioxide (CO_2_) have sparked widespread discussions on climate warming^1,2^. Theoretically, globally-averaged total precipitable water (TPW) should increase as well as warmer air has a higher capacity for holding moisture, under the assumption of constant relative humidity. Higher amounts of moisture in the atmosphere can increase global precipitation^3^, although the global response may be complex and dependent on surface type^4^. Previous studies of atmospheric temperature trends based on analysis of satellite measurements indicated signals for warming in the troposphere^5^ and cooling in the stratosphere^6,7^. Fu et al.^6^ analyzed a time series of Microwave Sounder Unit (MSU) channel 4 brightness temperatures at 57.95 GHz and found cooling in the stratosphere. The four MSU channels centered at 50.30, 53.74, 54.96, and 57.95 GHz correspond to temperatures of the Earth’s surface, lower troposphere, middle troposphere, and stratosphere, respectively. Spencer and Christy^5^ analyzed 10 years (1979 to 1988) MSU brightness temperatures (BT) at 53.74 GHz (channel 2) and detected global warming at the surface and in the lower troposphere. Vinnikov and Grody^8^ analyzed 24 years of MSU data from the NOAA series of polar-orbiting satellites and found a positive trend of 0.24 K/decade, consistent with observed global warming trends from ground observations. The uncertainty in the trend calculation is mainly caused by differences in sensors, satellites, and satellite local overpass times, as well as changes in calibration algorithms^8,9^. Although all MSU sensors have the same central frequencies at their four channels, their spectral response functions, non-linearities of the sensor response, antenna efficiency and emissivity are slightly different. The antenna emission itself depends on the shelf temperature of each satellite. These differences can lead to errors in sensor data records because calibration algorithms cannot be perfect. The global mean value of observed brightness temperatures often displays local diurnal variations as well.

This study is limited to climatic scales of decades for the entire Earth. Twice data daily from a single satellite is good for studying the global trends of TPW and air surface temperature. For regional climate studies in a short-term temporal scale, higher-frequency data (such as hourly) is often preferred to capture important variability and trends over time. However, there is no single satellite that can observe our Earth more than twice everywhere. An accurate inter-satellite calibration is necessary and important for studying regional climate changes. More accurate reanalysis system is also required for assimilating multi-satellite observations along with the observational errors.

## Results

### Monthly time series of the global mean anomalies of TPW and surface temperature

In order to minimize the uncertainty due to instrument calibration changes over time, and differences between instruments (inter-sensor calibration), it is desirable for climate studies to use well-calibrated sensor data records (SDRs) from the same sensor on the same satellite with a fixed local equatorial crossing time. The reprocessed Suomi-NPP (S-NPP) Advanced Technology Microwave Sounder (ATMS)^10^ is such a data set with a state-of-art calibration algorithm for studying climate change. Additionally, atmospheric and surface variables including TPW and surface temperature are available from S-NPP ATMS using the Microwave Integrated Retrieval System (MiRS)^11^ since 2011.

**Fig. 1:**
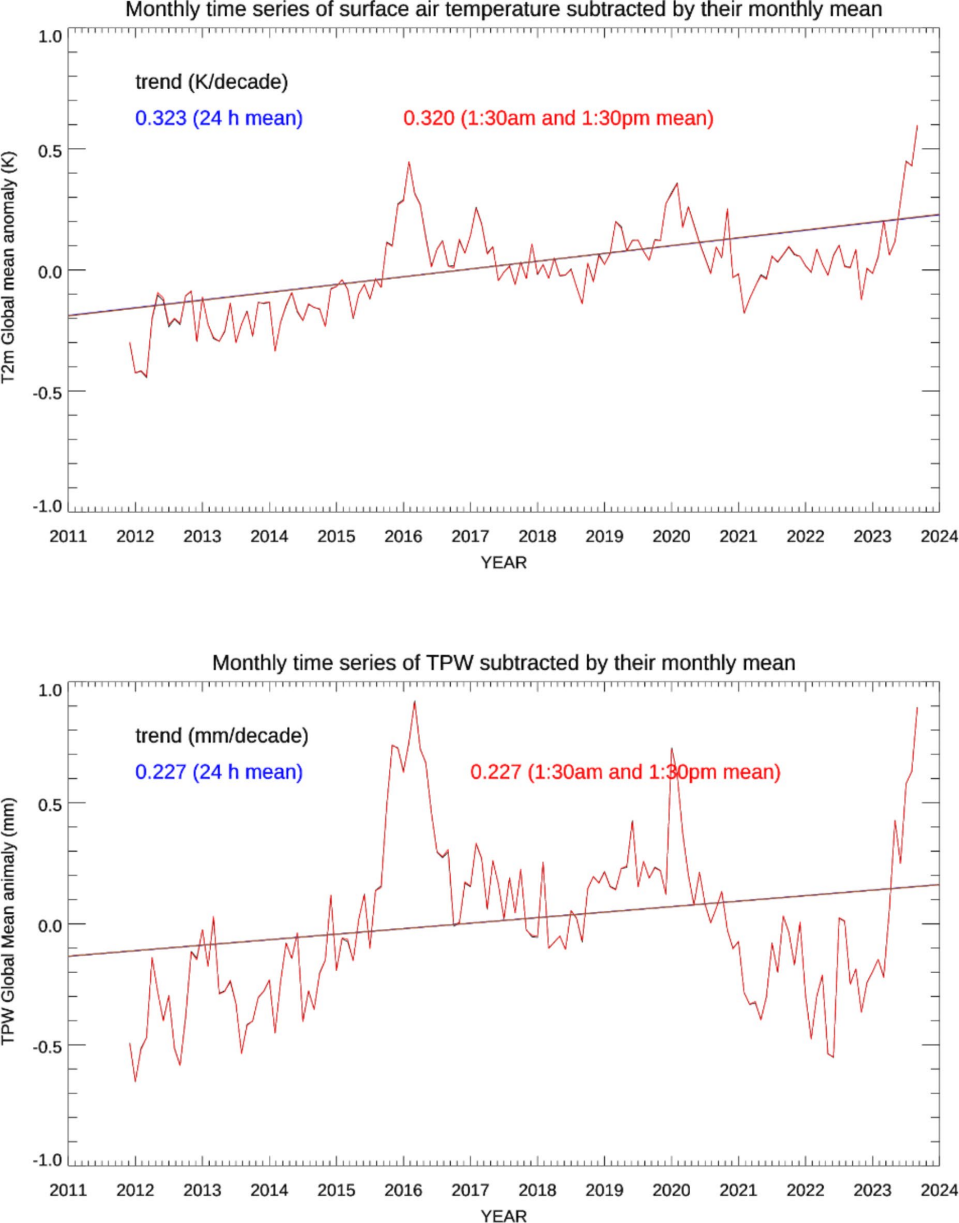
Time series of global monthly anomalies of 2-meter air temperature (top) and TPW (bottom) derived from ERA5 analyses. The straight lines are the linear trends derived from the monthly anomalies. Anomalies and trends are shown for global means based on 1:30 am/1:30 pm local time data only (red) and all hourly data (black). The anomalies and trends for both data sets are so similar that the black curves are almost obscured by the red curves. Computed annual trend values for both 24-hour and 1:30 am/pm data are shown in blue and red, respectively.

To answer the question of whether the true global mean trend can be captured by observations exclusively at approximately 1:30 am (S-NPP descending) and 1:30 pm (Suomi-NPP ascending) local times, we compared the monthly time series derived from 1:30 am and 1:30 pm (local time) ERA5 data against the monthly time series based on averaging over all ERA5 hourly data for TPW and surface air temperature at 2 m above the surface. About sixty-three and seven hundred fifty eight million data points were used to calculate the monthly global trend in the first and the second sample method, respectively. The ERA5 data provide hourly estimates of a large number of atmospheric, land and oceanic climate variables specified on a 0.25 degrees spatial resolution grid for both latitude and longitude since January 1940^12–14^. These hourly data are given at Coordinated Universal Time (UTC) and the data at 1:30 am and 1:30 pm local time are interpolated from the UTC times and longitudes. The ERA5 datasets contain accurate long-term records of the past global weather and have also served as a unique training dataset for the development of Artificial Intelligence (AI) weather forecast models: NVIDIA FourCastNet^15^, Pan-gu^16^, and Google DeepMind GraphCast^17^. Figure 1 shows a comparison for the global mean anomalies of TPW and 2-meter air temperature. The trends of the monthly time series based on 1:30 am/1:30 pm local times (red line) and based on all hourly data (black line) are practically the same. The black line is almost overlapped by the red line in Fig. 1. This suggests that the satellite observations at 1:30 am/1:30 pm local time can be used to reliably calculate the global monthly mean trend of TPW and air temperatures at 2 m above the surface. The prominent peaks of TPW and surface air temperature anomalies in Fig. 1 correspond to the very strong El Nino event in 2015–2016^18^.

We also studied the TPW trends by using ERA5 data based on 10:30 am/10:30 pm and 12:30 am/12:30 pm. The trends of the monthly time series are practically the same as the trend based on all hourly data. The monthly mean and globally spatial average may simplify diurnal variations so that one-time data during day and one-time data at night can represent the globally and monthly mean TPW trend. Large discrepancies among the TPW trends are found without using pm data.

### Increasing surface pressure

High concentrations of water vapor in the atmosphere are accompanied by a higher water vapor partial pressure. The water vapor pressure to the surface can be calculated from the mass in term of TPW. To calculate the trends of the surface pressure and its partitioning, we need to know the total mass of the atmosphere and the total surface area as well as to convert the masses of TPW and carbon dioxide (CO_2_) to their partial pressures. The global mean pressure at a sea-level is 1013.25 hPa. On some websites, the sea-level pressure was misinterpreted as the global mean surface pressure and total mass of the atmosphere was incorrectly computed by using the sea level pressure. The total mass of the atmosphere used on other websites appears to assume a range of values and different surface areas and various surface pressures may be used. The surface pressure in a paper from the 1980s^19^ was 984.4 hPa, using December 1979 through December 1985 ECMWF analyses. However, the accuracies of weather forecast and analyses have been greatly improved since then. We computed an updated surface pressure to be 985.5 hPa, using ERA5 data from December 1979 through December 1985. In this study, we calculate the mass and pressure using a hydrostatic equation. The surface pressure in Pa may be calculated as a gravity force divided by an area A:

1 $$P=\frac{mg}{A}$$

where m is the mass in kg and g equals to 9.80665 $m{s}^{-2}$ for a gravitational acceleration near Earth’s surface. The Earth’s surface area is 510.072 million square kilometers (https://en.wikipedia.org/wiki/Earth), which corresponds to an Earth radius of 6371.0469 km. Therefore, the total mass of the atmosphere (${M}_{atm}$) for a surface pressure of 98,570 Pa (i.e. 985.7 hPa, averaged from 2012 to 2022) is

2 $${M}_{atm}=510.072\times {10}^{12} \times \frac{98{,}570}{9.80665}=5.1267\times {10}^{18}\,kg$$

The mean TPW between 2012 and 2022 is 25 millimeters or 25 $kg\, {m}^{-2}$. The total mean mass of water vapor (${M}_{{H}_{2}\text{O}}$) can be calculated from the global and multiple year mean TPW:

3 $${M}_{{H}_{2}O}=25\,\text{k}\text{g}\,{m}^{-2}\times 510.072\times {10}^{12}\,{m}^{2}=12.751\times {10}^{15}\, kg$$

For a CO_2_ level of 414.70 Part per Million (PPM) in 2021 (https://gml.noaa.gov/webdata/ccgg/trends/co2/co2_annmean_gl.txt), the global total CO_2_ mass (${M}_{\text{C}{O}_{2}}$) in the atmosphere can be calculated as follows:

4 $${M}_{\text{C}{O}_{2}}=0.0004147\times \frac{44.087}{28.971}\times {(M}_{\text{a}\text{t}\text{m}}-{M}_{{H}_{2}O})=3.227\times {10}^{15}\, kg$$

Where 44.087 is the CO_2_ molar weight and 28.971 is the dry air molar weight. The value in the parenthesis of Eq. (4) is the total mass of dry air. The total CO_2_ pressure (${P}_{\text{C}{O}_{2}}$) to the surface can be estimated from a mass partition as:

5 $${P}_{\text{C}{O}_{2}}=\frac{g\times {M}_{\text{C}{O}_{2}} }{510.072\times {10}^{12}\,{m}^{2}}=62.05\,\text{P}\text{a}\approx 0.62\,\text{h}\text{P}\text{a}$$

Therefore, total CO_2_ in the atmosphere contributes 0.62 hPa to the total surface pressure.

**Fig. 2:**
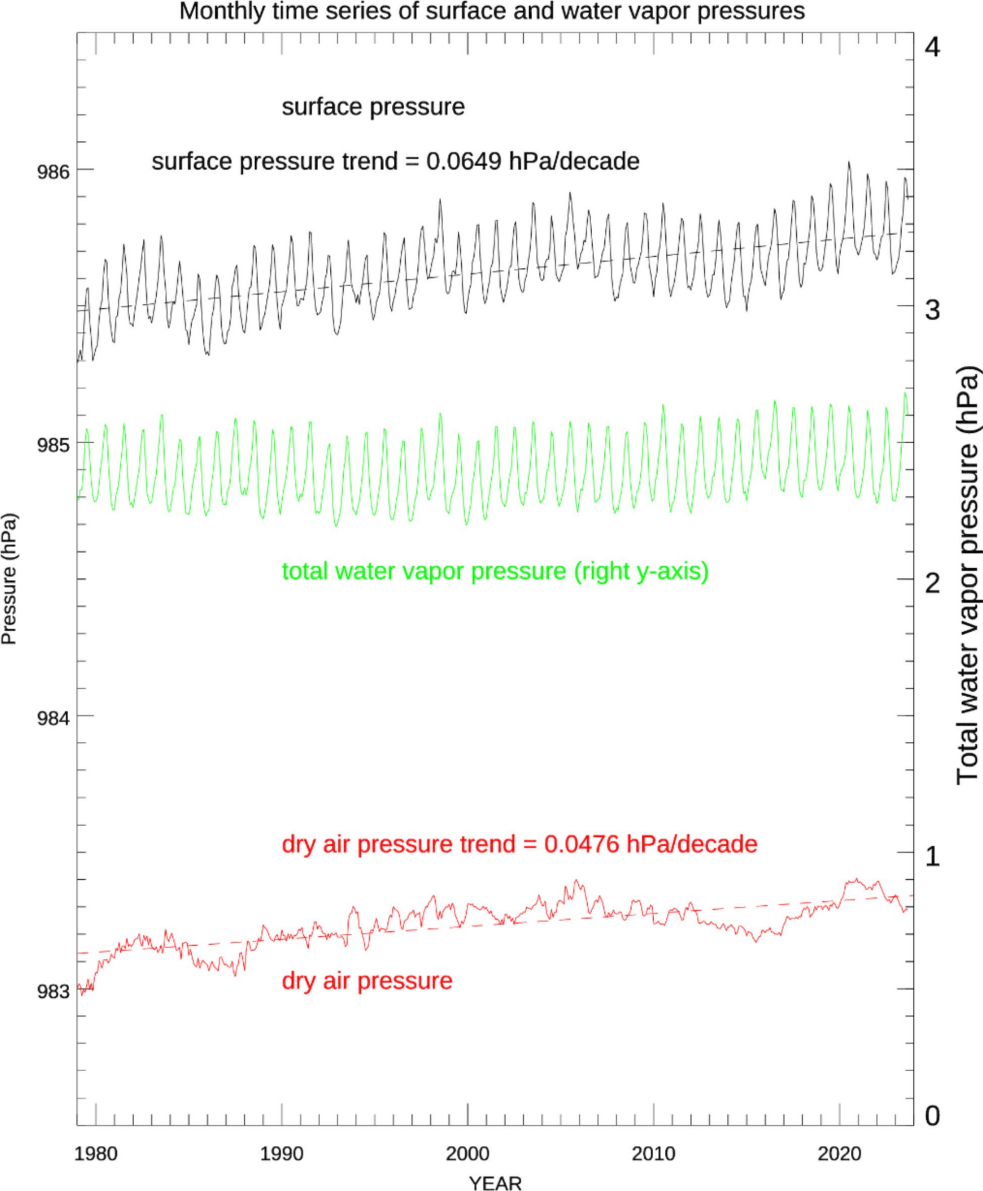
Time series of surface pressure based on ERA5 data, broken down into components: total surface pressure (black), the contribution of water vapor to total atmospheric pressure (green), the total dry air pressure (red), obtained by subtracting the water vapor pressure contribution from the total surface pressure. Dashed lines are the regressed trends of the corresponding variables.

As shown in Fig. 2, which is also derived from ERA5 data, the contribution of water vapor (and therefore TPW) to total surface pressure displays a strong seasonal variation (green line) with a maximum in July each year. The surface pressure (black line) shows a very similar seasonal variation as water vapor partial pressure displays (green line). The trend of the surface pressure is 0.0649 hPa/decade. Subtracting the water vapor pressure from the surface pressure eliminates the seasonal variation in surface dry pressure as indicated by the red line in Fig. 2. The global monthly mean of surface dry pressure should be almost constant, varying only with small changes in atmospheric composition. However, we found that the trend of the surface dry pressure is 0.0476 hPa/decade, which represents 73% of the trend for the total surface pressure. The increasing CO_2_ is a main contributor to the trend of the surface dry pressure and the total surface pressure, which is surprising. In the last decade (2012 to 2021), CO_2_ concentration has increased by about 24.48 PPM (https://gml.noaa.gov/ccgg/trends/gl_gr.html) roughly about 0.037 hPa/decade. The trend of CO_2_ as a fraction of the total surface dry pressure agrees with the trend of the surface dry pressure, which indicates that ERA5 surface pressure is consistent with both the TPW trend and the trend of CO_2_ on the surface pressure. We can conclude that the ERA5 data preserve the mass conservation of the atmosphere.

## Discussions

This study shows that the observations collected at 1:30 am and 1:30 pm local times can accurately reflect the global monthly mean trend for surface air temperature and total precipitable water. This study also delivers information to climate studies for a medium-term scale (decades) and a spatial scale of the entire Earth. The monthly mean here was calculated from 63 million ERA5 data points, twice times data each day. Sun-synchronous polar-orbiting satellite observing systems like S-NPP ATMS monthly provide about 90 million-point observations at 1:30 am and 1:30 pm local times about atmospheric temperatures and water vapors. The mean spatial resolution of S-NPP ATMS is comparable to the spatial resolution of ERA5 data. Therefore, the finding of this paper may indicate that the S-NPP ATMS observations can be used to independently study the monthly global trends of the TPW and air temperature. The finding provides a rationale to continue the operation of S-NPP in its current orbit with observations at the fixed local times.

## Electronic supplementary material

Below is the link to the electronic supplementary material.

Supplementary Material 1

## Data Availability

All data and codes for Figs. 1 and 2 will be uploaded to the journal data site. Please see a supplemental material file (shared_code_data.tar) or https://figshare.com/s/b9e486563e8cda20b119) on both submission system and in the manuscript.
