# Supplementary figures and images for: Trends of temperature and total precipitable water, as well as the trend of surface pressure induced by CO2
Source: Sci Rep. 2024 Nov 25;14:29202. doi: 10.1038/s41598-024-80685-8 (PMC11589833; doi:10.1038/s41598-024-80685-8)

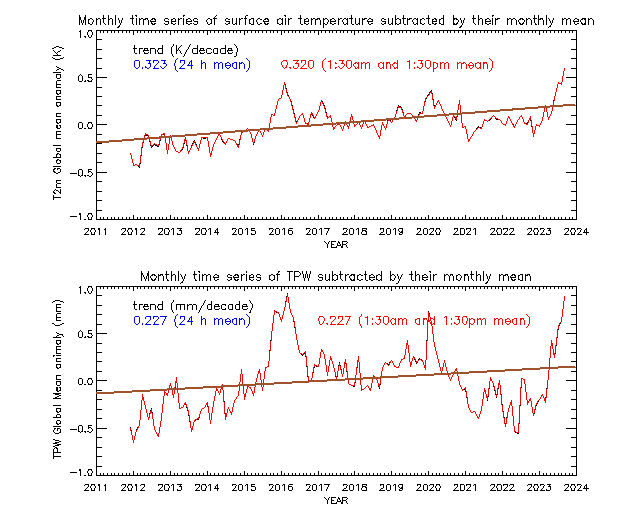

Supplement: Supplementary file 1 — Supplementary Material 1 [file 41598_2024_80685_MOESM1_ESM.tar › shared_code_data/Fig1.png]

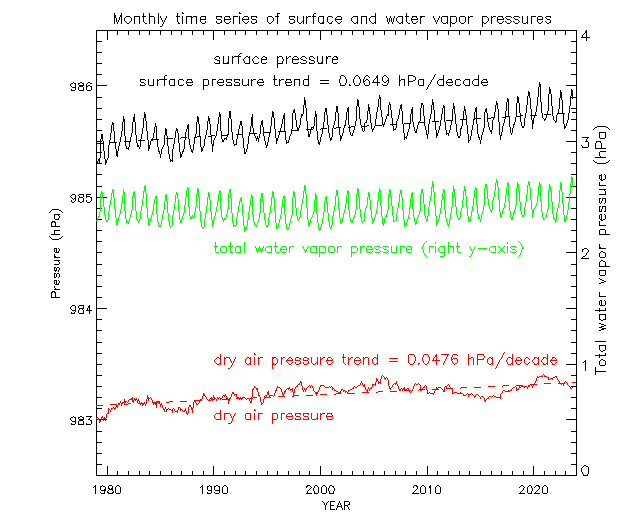

Supplement: Supplementary file 1 — Supplementary Material 1 [file 41598_2024_80685_MOESM1_ESM.tar › shared_code_data/Fig2.png]
